# Prediction of invasive candidal infection in critically ill patients with severe acute pancreatitis

**DOI:** 10.1186/cc12569

**Published:** 2013-03-18

**Authors:** Alison M Hall, Lee AL Poole, Bryan Renton, Alexa Wozniak, Michael Fisher, Timothy Neal, Christopher M Halloran, Trevor Cox, Peter A Hampshire

**Affiliations:** 1 Intensive Care Unit, Royal Liverpool University Hospital, Prescot Street, Liverpool, L7 8XP, UK; 2 Acute Medical Unit, Royal Liverpool University Hospital, Prescot Street, Liverpool, L7 8XP, UK; 3 Medical Microbiology Department, Royal Liverpool University Hospital, Prescot Street, Liverpool, L7 8XP, UK; 4 NIHR Pancreas Biomedical Research Unit, Department of Molecular and Clinical Cancer Medicine, Institute of Translational Medicine, University of Liverpool, Daulby Street, L69 3GA, UK; 5 Department of Biomedical Statistics, University of Liverpool, Daulby Street, L69 3GA, UK

## Abstract

**Introduction:**

Patients with severe acute pancreatitis are at risk of candidal infections carrying the potential risk of an increase in mortality. Since early diagnosis is problematic, several clinical risk scores have been developed to identify patients at risk. Such patients may benefit from prophylactic antifungal therapy while those patients who have a low risk of infection may not benefit and may be harmed. The aim of this study was to assess the validity and discrimination of existing risk scores for invasive candidal infections in patients with severe acute pancreatitis.

**Methods:**

Patients admitted with severe acute pancreatitis to the intensive care unit were analysed. Outcomes and risk factors of admissions with and without candidal infection were compared. Accuracy and discrimination of three existing risk scores for the development of invasive candidal infection (Candida score, Candida Colonisation Index Score and the Invasive Candidiasis Score) were assessed.

**Results:**

A total of 101 patients were identified from 2003 to 2011 and 18 (17.8%) of these developed candidal infection. Thirty patients died, giving an overall hospital mortality of 29.7%. Hospital mortality was significantly higher in patients with candidal infection (55.6% compared to 24.1%, *P *= 0.02). *Candida *colonisation was associated with subsequent candidal infection on multivariate analysis. The Candida Colonisation Index Score was the most accurate test, with specificity of 0.79 (95% confidence interval [CI] 0.68 to 0.88), sensitivity of 0.67 (95% CI 0.41 to 0.87), negative predictive value of 0.91 (95% CI 0.82 to 0.97) and a positive likelihood ratio of 3.2 (95% CI 1.9 to 5.5). The Candida Colonisation Index Score showed the best discrimination with area under the receiver operating characteristic curve of 0.79 (95% CI 0.69 to 0.87).

**Conclusions:**

In this study the Candida Colonisation Index Score was the most accurate and discriminative test at identifying which patients with severe acute pancreatitis are at risk of developing candidal infection. However its low sensitivity may limit its clinical usefulness.

## Introduction

Infections caused by fungal pathogens have increased in the last two decades with data from the USA between 1979 and 2000 demonstrating a 207% increase [1]. In the Extended Prevalence of Infection in Intensive Care (EPIC II) study, candida was the fourth most common cause of infection in ICUs worldwide [2]. Infections in Europe are less frequent; however, in the Sepsis Occurrence in Acutely Ill Patients (SOAP) study, candidal organisms still accounted for 17% of infections [3]. Other data demonstrate that candidaemia in intensive care patients, however, has remained static or even decreased in recent years [4]. Most cases are caused by *Candida albicans*, but there are numerous other species, and antifungal resistance spectrums of these vary.

Numerous risk factors have been identified for the development of C*andida spp *bloodstream infection. These include the presence of invasive lines, for example central venous catheters (CVCs), antibiotic therapy, acute kidney injury requiring renal replacement therapy (RRT), malignancy and neutropenia, previous abdominal surgery, total parenteral nutrition (TPN), long term hospitalisation and prior fungal colonisation [5-8].

Severe acute pancreatitis (SAP) has also been identified as a risk factor for candidal infection [9]. Candidal infection has been identified as a cause of increased mortality in patients post-operatively and in the critically ill [8,10,11] but its effect on the outcome of SAP has been disputed [12-15]. A healthy pancreas is relatively resistant to fungal infection; however, pancreatic necrosis carries a disproportionately higher risk of infection with bacterial and fungal organisms. Prior use of antibiotics has been demonstrated to increase the risk of fungal infection [5]. Prophylactic or empirical antifungal treatment has been advocated for high-risk surgical patients [16-19] and demonstrated to prevent fungal infection in SAP patients [12]. 'Prophylactic' antifungal treatment is defined as administration of antifungals to patients identified as having a particular diagnosis or particular factors that confer a high risk of subsequent fungal infection. 'Empirical' treatment is defined as antifungal therapy given to patients with clinical features of an inflammatory response consistent with infection but without microbiological confirmation. As delays in treatment are associated with increased mortality and fungal culture can take up to 72 hours, it would be desirable to identify patients at risk for invasive infection whilst minimising unnecessary treatment and reducing the risk of resistance through increases in non-albicans species [7,20].

As a consequence, several risk scoring systems have been developed in an attempt to aid discrimination between candida colonisation and invasive candidal infection (ICI) [6,8,9]. These combine the identification of high-risk patients with clinical and microbiological data to identify those patients at risk of developing ICI. The aim of this study was to identify the prevalence of ICI in a population of critically ill patients with SAP, to identify risk factors for the development of ICI and to evaluate its impact on patient outcome. In addition we assessed the accuracy and discrimination of three previously described risk scores for ICI in this cohort of patients [6,8,9].

## Materials and methods

A single centre, retrospective study was conducted at a tertiary referral centre for patients with SAP. After discussion with the local Research Ethics Committee, the study protocol was approved and the requirement for written informed consent was waived, since informed consent for collection and analysis of patient data recorded in the Case Mix Programme database is not required under Section 251 of the NHS Act 2006 (approval number PAIG 2-10(f)/2005). All patients admitted to the ICU with a diagnosis of SAP between July 2003 and February 2011 were screened for inclusion in the study. Suitable cases were identified from the admissions database. Patients who were re-admitted to the ICU during the same hospital stay or who were transferred from other ICUs were excluded from further analysis. SAP was defined as acute pancreatitis with true organ dysfunction irrespective of local complications as per consensus guidelines in 2004 [21]. 'Significant necrosis' was defined as the presence of more than 30% necrosis seen on abdominal computed tomography scans, as reported by a radiologist. Three independent workers extracted data from the case notes and electronic records of each admission onto an Excel spreadsheet. The aetiology of SAP was classified into 'alcohol', 'gallstones', 'drug', or 'idiopathic'. 'Unknown' was recorded if no cause could be identified from the case notes. Acute Physiology and Chronic Health Evaluation (APACHE II) scores were extracted from the ICU admission database. For admissions without APACHE II scores due to a unit length of stay less than eight hours, admission physiology data were entered into a web-based calculator [22] in order to calculate an admission APACHE II score.

All patients were treated according to a routine standard of care. This included adherence to care bundles, no prophylactic antibiotics, daily measurement of C-reactive protein and early enteral feeding. Naso-gastric feeding was used for nutritional support, guided by the patient's ideal body weight. Prokinetics were started when absorption was poor and, if necessary, a post-pyloric feeding tube was inserted. TPN was considered if post-pyloric feeding was unsuccessful. Abdominal computed tomography scans were performed on admission and then every seven to ten days, and minimally invasive pancreatic necrosectomy (MIRPN), open necrosectomy or radiologically-guided drainage was performed, as surgically indicated.

Samples for candidal colonisation were taken routinely from tracheal aspirates and/or bronchial lavage, skin swabs and drainage fluid (if drains were *in situ*). Further sampling (for example, culture of blood, line tips and pancreatic tissue samples) was performed if indicated by clinical need. Patients were classified as having ICI if they had: 1) ≥ 1 positive blood culture or 2) ≥ 1 positive pancreatic tissue culture or 3) ≥ 1 pancreatic drain fluid culture positive for *Candida *spp. and in addition received antifungal drugs after the positive drain fluid culture. Antifungal therapy is only initiated after discussion between an intensive care consultant and a medical microbiologist with an interest in intensive care. Patients with *Candida *cultured from one or more samples from respiratory secretions, urine, line tips, or wound or skin swabs alone were classified as colonised.

For determination of risk in the scoring systems, the following were considered risk factors: 1) severe sepsis as defined by the 2001 SCCM/ESICM/ACCP/ATS/SIS International Sepsis Definitions Conference criteria [23]; 2) central venous access: presence of a CVC or peripherally inserted central venous catheter (PICC) on days one to three post ICU admission; 3) systemic antibiotics: any intravenous antibiotics on days one to three post ICU admission (excluding prophylactic antibiotics given after surgery or to treat reduced gastric motility); 4) renal replacement therapy (RRT): any form of RRT given on day one to three post ICU admission; 5) steroids: any dose of corticosteroids given during the seven days prior and three days after admission to ICU; 6) immunosuppressive drugs: any immunosuppressive drugs (as listed in the British National Formulary version 59 section 8.2) given during the seven days prior to admission; 7) surgery: any intra-abdominal surgical procedure during the seven days prior to admission; and 8) TPN: any TPN delivered via a CVC or PICC on days one to three post ICU admission.

### Statistics

Descriptive statistics were performed on the entire cohort of admissions and on admissions with and without ICI. Univariate analysis was performed to assess differences in the characteristics of patients with and without ICI. ICU and ultimate hospital mortality were compared between the two groups.

Multivariate logistic regression analysis was performed using ICI as the dependent variable with candida colonisation, presence of necrosis and length of ICU stay as independent variables.

'Candida score' was calculated for each admission as described previously [6] with appropriate weightings for each variable. Weightings are as follows: TPN, surgery, multifocal colonisation: 1 point; severe sepsis: 2 points. Patients with a score >3 were defined as 'positive'. Since the Invasive Candidiasis score includes SAP as a risk factor, SAP was removed for this study and a modified 'Invasive Candidiasis' risk score [9] was calculated for each admission as follows: Patients who had developed ICI and met the criteria of having received antibiotics and had a CVC and at least one of: TPN, RRT, surgery, steroids or immunosuppressants were defined as true positives. Immunosuppression was defined as described above. A Candida Colonization Index Score (CCIS) was calculated for each patient using the methods described [8] as follows: CCIS = ratio of the number of non-blood distinct body sites colonised with *Candida spp *to the total number of body sites cultured. A CCIS ≥ 0.5 predicts *Candida *infection; therefore, patients who had ICI and a CCIS ≥ 0.5 were defined as true positives.

The sensitivity, specificity, positive predictive value (PPV), negative predictive value (NPV), positive likelihood ratio (LR+) and negative likelihood ratio (LR-) were calculated for each score, along with 95% confidence intervals. For PPV and NPV, prevalence levels as described in the study population were used. Area under the Receiver Operating Characteristic curve (AUROC) with exact binomial confidence intervals was calculated for each score using the method of DeLong [24]. Comparisons between groups of categorical data were made using Fisher's exact test or χ^2 ^test where appropriate. Continuous data were compared using the student's t test for normally distributed data, or the Mann-Whitney U test for non-normally distributed data. Results were considered statistically significant when *P *values were <0.05. MedCalc v.12.0 (MedCalc Software, Belgium) was used for statistical analysis.

## Results

**Figure 1:**
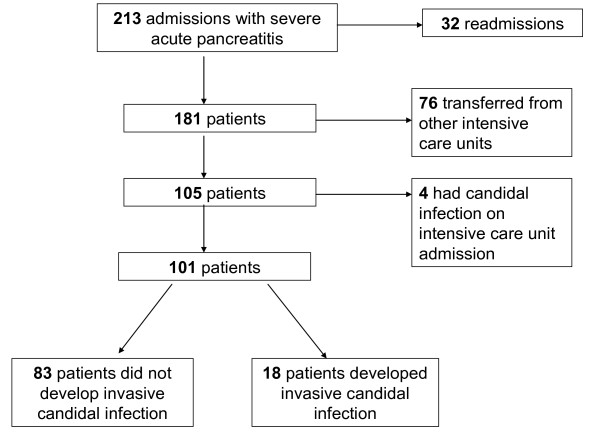
**Consort diagram for recruitment of patients**.

There were 213 ICU admissions with SAP during the study period. There were 32 re-admissions of 26 patients during the same hospital stay. Seventy-six patients were excluded as they were directly transferred to the ICU from other hospitals. Four patients already had established candidal infection on admission to ICU and were excluded from further analysis (Figure 1). Therefore, 101 patients were included of whom 58 (57%) were men. The median (IQR) age was 60 (50 to 73) years, and the most common causes of SAP were gallstones (45 (44.6%)) and alcohol (30 (29.7%) (Table 1)

**Table 1:** Baseline clinical data from all patients

	All patients	Patients without invasive candidal infection	Patients with invasive candidal infection	*P *value
Number of patients (%)	101	83 (82.2)	18 (17.8)	
Age, years (median, IQR))	60 (50 to 73)	60 (50-72)	62.5 (44.75 to 76.25)	0.884
Median LOS ICU (days, IQR, range)	8.9 (3.5 to 17.3, 0.1 to 53.1)	7.3 (3.1 to 14.7, 0.1 to 53.2)	16.9 (9.4 to 23.0, 2.2 to 53.1)^a^	0.004
Male gender (%)	58 (57.4)	48 (57.8)	10 (55.6)	0.999
Median hospital LOS prior to ICU (days, IQR, range)	4 (1 to 12, 0 to 110)	4 (1 to 12, 0 to 110)	4.5 (2 to 13, 0 to 17)	0.125
APACHE II (median, IQR)	16 (12 to 21)	16 (12 to 20.5)	17 (15 to 21.75)	0.445
Mortality, ICU number (%)	18 (17.8)	13 (15.7)	5 (27.8)	0.305
Hospital number (%)	30 (29.7)	20 (24.1)	10 (55.6)^a^	0.020
Surgery, number (%)	29 (28.7)	22 (26.5)	7 (38.9)	0.389
Severe sepsis, number (%)	37 (36.6)	30 (36.1)	7 (38.9)	0.999
Significant pancreatic necrosis, number (%)	53 (52.5)	40 (48.2)	13 (72.2)	0.063
Pancreatic intervention, number (%)	MIRPN 27 (26.7)	23 (27.7)	4 (22.2)	0.774
	Open necrosectomy 18 (17.8)	11 (13.3)	7 (38.9)^a^	0.017
	Radiologically-guided drain 5 (5)	4 (4.8)	1 (5.6)	0.999
	Unknown 7 (6.9)	6 (7.2)	1 (5.6)	0.999
	No intervention 44 (44)	39 (47)	5 (27.8)	0.191
Screened for colonisation with candida, number (%)	95 (94.1)	77 (92.8)	18 (100)	0.588
Total body sites screened	340	269	71	
Total sites positive for Candida	107	67	40	
Proportion of sites positive to sites screened	0.31	0.25	0.56	
Aetiology number (%)	Gallstones 45 (44.6)	38 (45.8)	7 (38.9)	0.794
	Alcohol 30 (29.7)	25 (30.1)	5 (27.8)	0.999
	Drugs 1 (1)	0 (0)	1 (5.6)	0.178
	Other 10 (9.9)	7 (8.4)	3 (16.7)	0.378
	Idiopathic 3 (3.0)	2 (2.4)	1 (5.6)	0.081
	Unknown 12 (11.9)	10 (12)	2 (11.1)	0.999

^a^*P *<0.05. APACHE II, Acute Physiology and Chronic Health Evaluation II; LOS ICU, length of stay in the ICU; MIRPN, minimally invasive retroperitoneal pancreatic necrosectomy.

There was no significant difference in APACHE II scores between the two groups. Eighteen (17.8%) patients developed ICI. Patients with ICI had a longer median length of ICU stay (16.9 versus 7.3 days, *P *= 0.0043). There was a significant association between open necrosectomy and subsequent ICI (Table 1, *P *= 0.0171) on univariate analysis, but this was not significant in regression analysis. Overall, 18 (17.8%) patients died in ICU with a higher mortality in patients with ICI (5/18 (27.8%) versus 13/83 (15.7%)). Overall hospital mortality was 29.7% (30/101) which was significantly higher in patients who developed ICI: 10/18 patients (55.6%) died, compared to 20 deaths in 83 patients without ICI (24.1%) (*P *= 0.0201) (Table 1).

Table 2 displays the risk factors for development of ICI for those patients with and without ICI. There were no significant differences in incidence of severe sepsis, or use of CVC lines, antibiotics, RRT, steroids, immunosuppressive therapy, previous surgery or TPN between the two groups. Of the known risk factors, only colonisation with *Candida *spp. was significantly greater in the ICI group. Sixteen (88.9%) patients with invasive candida infection were colonised with candida, as opposed to 37 (44.6%) without subsequent infection (*P *= 0.0006) (Table 2). Using logistic regression analysis, colonisation with candida (OR 4.33) was the only factor significantly associated with invasive candidal infection (Table 3).

**Table 2:** Risk factors for the development of invasive candidal infection

	Patients without invasive candidal infection	Patients with invasive candidal infection	*P *value
Patients, number	83	18	
Severe sepsis on admission to ICU, number (%)	30 (36.1)	7 (38.9)	0.999
CVC, number (%)	74 (89.2)	17 (94.4)	0.686
Antibiotics, number (%)	53 (63.9)	14 (77.8)	0.409
Renal replacement therapy, number (%)	14 (16.9)	4 (22.2)	0.734
Steroids, number (%)	34 (41)	7 (38.9)	0.999
Immunosuppressive drugs, number (%)	2 (2.4)	2 (11.1)	0.145
Surgery, number (%)	22 (26.5)	7 (38.9)	0.389
TPN, number (%)	26 (31.3)	8 (44.4)	0.288
Significant pancreatic necrosis, number (%)	40 (48.2)	13 (72.2)	0.063
Positive blood culture, number (%)	28 (33.7)	8 (44.4)	0.424
Colonised with candida, number (%)	37 (44.6)	16 (88.9)	>0.001

CVC, central venous catheter; ICU, Intensive Care Unit; TPN, total parenteral nutrition.

**Table 3:** Multivariate logistic regression analysis of risk factors for ICI for patients with SAP admitted to ICU

	Odds ratio (95% CI)	*P *value
Colonisation with candida	4.33 (1.07 to 17.5)	0.04
ICU length of stay	1.01 (0.96 to 1.06)	0.721
Significant necrosis	0.36 (0.10 to 1.29)	0.118

SAP, severe acute pancreatitis.

### Candida infections

Eighteen patients developed ICI, giving an infection rate of 13.2 per 1,000 days (18/1,359 days). Candidaemia was present in 5 (27.8%) infected patients (3.7 per 1,000 days). Five patients had only candidaemia, whereas three patients with candidaemia also had either tissue or abdominal fluid samples that were positive for *candida *spp. *Candida *spp. were isolated in pancreatic tissue in four patients. Ten patients had *Candida *in abdominal drain fluid samples only and received antifungal medication.

### Candida species isolated

**Figure 2:**
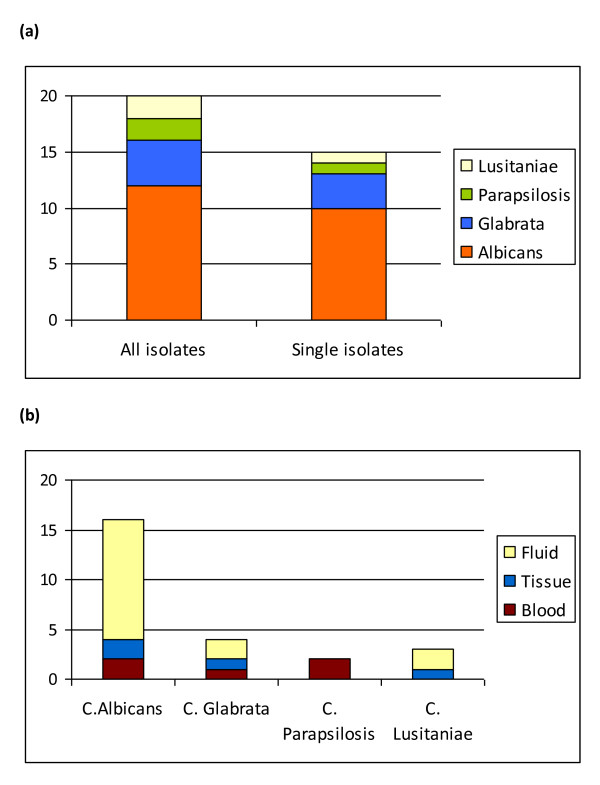
**Candidal species isolated**. **(a) **The number of candidal species isolated from patients. The left hand bar shows the contribution of candidal species isolated from patients, including mixed growth; the right hand bar shows single isolates only. **(b) **The proportion of each candidal species cultured from all positive isolates in patients classified as infected with candida.

In the patients with a positive blood or tissue culture, *C. albicans *was isolated in seven patients, *C. glabrata *and *C. lusitaniae *each in one patient and *C. albicans and C. glabrata *in one patient. In patients who had a positive drain fluid culture and subsequent antifungal therapy, there were three patients with *C. albicans*, two with *C. glabrata *and one each with *C. parapsilosis *and *C. lusitaniae*. One sample contained a mixed growth of *C. albicans *and *C. parapsilosis *(Figure 2a and 2b).

### Candida risk scores

Data regarding colonisation screening were not available for six patients and so these were excluded from analysis of the performance of the CCIS. The risk prediction scores tested demonstrated low sensitivities, with values below 0.7 (Table 4). The Candida Score had the highest specificity of 0.85 and the CCIS had a specificity of 0.79. All scoring systems had high NPVs (>0.7). PPVs were all below 0.5. The CCIS demonstrated a LR + of 3.2 and the other scores tested had lower LR + values. No scores had LR - below 0.1.

**Table 4:** Comparison of the diagnostic accuracy and discrimination of the Candida score, Modified Invasive Candidiasis Score and Candida Colonisation Index score in predicting ICI

	**Candida Score **[6]	**Modified Invasive Candidiasis score **[9]	**Candida Colonisation Index score **[8]
Sensitivity (95% CI)	0.23 (0.10-0.42)	0.61 (0.36-0.83)	0.67 (0.41-0.87)
Specificity (95% CI)	0.85 (0.74-0.92)	0.49 (0.38-0.61)	0.79 (0.68-0.88)
Positive predictive value (95% CI)	0.39 (0.17-0.64)	0.21 (0.11-0.34)	0.43 (0.24-0.63)
Negative predictive value (95% CI)	0.72 (0.61-0.82)	0.85 (0.72-0.94)	0.91 (0.82-0.97)
Likelihood Ratio + (95% CI)	1.5 (0.7-3.5)	1.2 (0.8-1.9)	3.2 (1.9-5.5)
Likelihood Ratio - (95% CI)	0.9 (0.7-1.1)	0.8 (0.4-1.5)	0.4 (0.2-0.8)
Area under the ROC curve (95% CI)	0.62 (0.52-0.71)	0.59 (0.49-0.69)	0.79 (0.69-0.87)

**Figure 3:**
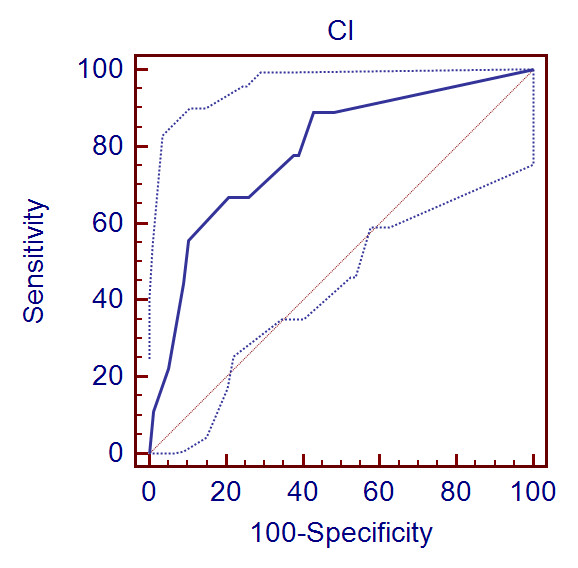
**AUROC for discrimination of the Candida Colonisation Index score with 95% confidence intervals (dotted lines)**.

The CCIS had the best discrimination of the scores tested, with AUROC of 0.79 (Figure 3). The other two scores demonstrated poor discrimination, with AUROCs less than 0.7.

## Discussion

An increase in Candida infection in non-neutropenic critically ill patients has been demonstrated to put them at increased risk of mortality and morbidity [4,6]. Whilst there is a concern that this is the case in patients with SAP [10,11,25-28], this has not been universally demonstrated [12-15]. It is, however, likely that colonisation plays an important instigating role in these invasive infections. Patients with SAP are at particular risk of ICI. In this study both colonisation with *Candida spp*. and a CCIS >0.5 were associated with subsequent infection [8]. It can be difficult to distinguish colonisation from ICI. There is evidence that delaying antifungals in ICI is associated with increased mortality [29]; however, current antimicrobial culture techniques can still take up to 72 hours to grow yeasts [30]. Therefore, the unanswered question is whether critically ill patients with SAP should receive routine prophylactic antifungals with the risk of selecting out resistant strains of candida or whether treatment should be delayed until a positive culture is obtained.

### Aetiology and infection

The aetiology of SAP in this study was predominantly gallstones (44%) or alcohol (30%). In this study, as in previous studies, there was no difference in the incidence of ICI in patients with either aetiology [31].

Our data indicate that a clinically significant proportion of patients (17.8%) with SAP develop ICI during their ICU admission, giving an infection rate of 13.2 per 1,000 days for ICI and 3.7 per 1,000 days for candidaemia. However, as our data includes infections other than candidaemias, the infection rate is higher than others have demonstrated [7,32,33]. ICI was associated with both a longer ICU length of stay and higher hospital mortality. APACHE II scoring did not distinguish between the two groups (median APACHE II scores were 17 and 16 for patients with and without ICI, respectively). Other groups have identified an association in critically ill patients between severity of illness and prevalence of ICI [8,11]. It might be expected that the prevalence of candidal infection should be higher in those with a greater severity of illness. Pittet *et al*. found that an APACHE II score of 20 or above was associated with increased nosocomial infections [8]. Our patients had a lower median APACHE II score than described in other studies that included critically ill patients with fungal infections, which may partly explain why we did not find the same association with candidal infection.

### Risk factors for invasive candidal infection

Previously identified risk factors for the development of fungal infection in critically ill patients include the presence of CVCs, the use of antibiotics and steroids, recent abdominal surgery and TPN [5-7,34]. In this study only colonisation with candida was identified as being significantly associated with subsequent candidal infection. Although we did not find an association between abdominal surgery prior to admission and candidal infection, there was a significant association between open necrosectomy and subsequent ICI on univariate analysis (Table 1). However, since open necrosectomies are performed less frequently and are reserved for more complex and difficult cases, this finding should be interpreted with caution since it may well represent a confounding factor. Indeed, on logistic regression analysis, open necrosectomy was not associated with candidal infection (Table 3). Antibiotics, steroids, CVC, TPN, RRT, immunosuppression and previous abdominal surgery were not associated with ICI in this study. In previous studies, the use of broad-spectrum antibiotics has been consistently associated with candidal infection [8,27,28,34,35]. One explanation for this discrepancy may be that we only looked at a period of three days after ICU admission when antibiotics were used and not the period before ICU admission.

Our data differ from those of other groups who have consistently demonstrated surgery, TPN and severe sepsis to be predictors of candidal infection in an unselected group of critically ill patients [6,34]; however, the number of patients who developed ICI in this study was small. More than 90% of our cohort of patients had central venous access and, therefore, it is perhaps not surprising that this did not discriminate between patients with and without ICI.

### Colonisation

Leon *et al*. demonstrated that multifocal colonisation (two or more non-contiguous sites) was a predictor of ICI [6]. The results of this study support the finding that colonisation is a risk factor for later infection with an odds ratio of 4.49 (Table 3). Simple numerical number of colonisation sites has been disputed as a technique sophisticated enough to predict invasive infection; and Pittet *et al*. developed a 'corrected colonisation index' that expressed the intensity of colonisation rather than just the number of sites [8]. A threshold index of 0.5 (positive/sites tested) was able to identify patients who went on to develop invasive bloodstream or other sterile site infection [8]. Our data demonstrated a significant association between candida colonisation and development of ICI, when compared to patients without ICI (88.9% versus 44.6% *P *= 0.006).

The CCIS had a NPV of 0.91 (Table 4) and specificity of 0.79, that is, patients with a CCIS <0.5 are unlikely to have invasive infection, although the negative LR was 0.4, above the value of 0.1 that has been suggested as being useful at the bedside [36]. The CCIS may be the most useful test to identify those patients who are unlikely to benefit from antifungal therapy. In these patients, the risks of administering prophylactic antifungals may outweigh the benefits. Using this rule in this cohort of patients, 28 (27.7%) would have received prophylactic antifungals, 16 of whom were 'false positives', and six patients would not have received prophylactic antifungals when they actually had an invasive candidal infection.

### Performance and discrimination of risk scores for invasive candidal infection

The AUROC is a measure of discriminative power with values above 0.8 suggesting excellent discrimination [37]. The CCIS was the most discriminating test between patients with invasive infection and those without, as assessed by the AUROC, with an AUROC of 0.79 (95% CI 0.69-0.87) (Figure 3). The other two scores did not show good discrimination in this cohort of patients (Table 4).

Although the PPV (0.43) and LR+ (3.2) were greatest for the CCIS among the three scores tested, they are not high enough to be clinically useful [36]. In agreement with other studies [37], the existing scoring systems are good at identifying those at lower risk of developing invasive candidal disease, since all scores showed reasonable specificities and NPV (Table 4).

### Limitations

This study is limited in its retrospective nature with the risk of missing data. True ICI is also difficult to identify. Our definition of ICI in those patients who had candida in abdominal fluid and received antifungal treatment could be disputed, as it may not be a completely accurate indicator of invasive disease. However, given the retrospective nature of this study, it is an appropriate assumption that patients who received antifungal therapy after a positive fluid culture represented true infection.

Another weakness in this study is that routine surveillance swabs of patients with SAP are not taken, so it could be argued that the association between colonisation and infection is not causative, since patients without signs of infection are less likely to have been screened. However, there was no significant difference in the proportion of patients with ICI who were screened (100%) and those without ICI who were screened (92.8%, *P *= 0.587). Patients known to be colonised with candida who then cultured candida in abdominal fluid may have been more likely to receive antifungal treatment, representing a confounding factor, despite the input from a medical microbiologist. The data may also be skewed by our hospital being a tertiary referral centre and receiving patients at varying stages of their disease. Finally, the study was conducted at a single-centre, and so our results may not be applicable to other health-care settings.

## Conclusions

Patients with SAP are known to be at high risk for ICI. We have demonstrated that ICI in SAP patients is associated with increased hospital mortality and longer duration of ICU stay. We have also shown that one of the existing risk scoring systems (CCIS [8]) in a population of critically ill patients with SAP has good discrimination to identify patients who are at low risk of developing ICI. Patients who have a CCIS of <0.5 are unlikely to go on to develop invasive candida infections. Further studies investigating the benefit of prophylactic antifungal treatment in patients with SAP and a CCIS of ≥ 0.5 are needed.

## Key messages

• Patients with pancreatitis and invasive fungal infection have significantly greater hospital mortality.

• Existing scoring systems are good at discriminating patients at low risk of developing invasive candidal infection.

• Colonisation significantly increases the risk of invasive infection.

• Patients with a CCIS of <0.5 are unlikely to develop invasive infection.

## Abbreviations

APACHE: Acute Physiology and Chronic Health Evaluation; CCIS: Candida Colonisation Index Score; CVC: central venous catheter; ICI: invasive candidal infection; LR: likelihood ratio; MIRPN: minimally invasive pancreatic necrosectomy; NPV: negative predictive value; PICC: peripherally inserted central catheter; PPV: positive predictive value; RRT: renal replacement therapy; SAP: severe acute pancreatitis; TPN: total parenteral nutrition; 95% CI: 95% confidence interval.

## Competing interests

The authors declare that they have no competing interests.

## Authors' contributions

AH carried out the data collection, statistical analysis and manuscript drafting. LP participated in the study design, data collection and manuscript drafting. BR participated in the study design and data collection. AW carried out data collection. MF carried out the microbiological results collection and data collection. TN participated in study design and microbiological results collection. CH contributed to the discussion and statistical analysis and critically reviewed the manuscript. TC provided statistical advice. PH conceived the study and participated in its design, data collection, statistical analysis and manuscript drafting. All authors read and approved the final manuscript.

## References

[B1] Martin GS Mannino DM Eaton S Moss M The epidemiology of sepsis in the United States from 1979 through 2000 N Engl J Med 2003 348 1546 1554 10.1056/NEJMoa022139 12700374

[B2] Vincent JL Rello J Marshall J Silva E Anzueto A Martin CD Moreno R Lipman J Gomersall C Sakr Y Reinhart K EPIC II Group of Investigators International study of the prevalence and outcomes of infection in intensive care units JAMA 2009 302 2323 2329 10.1001/jama.2009.1754 19952319

[B3] Vincent JL Sakr Y Sprung CL Ranieri VM Reinhart K Gerlach H Moreno R Carlet J Le Gall JR Payen D Sepsis Occurrence in Acutely Ill Patients Investigators Sepsis in European intensive care units: results of the SOAP study Crit Care Med 2006 34 344 353 10.1097/01.CCM.0000194725.48928.3A 16424713

[B4] Trick WE Fridkin SK Edwards JR Hajjeh RA Gaynes RP National Nosocomial Infections Surveillance System Hospitals Secular trend of hospital-acquired candidemia among intensive care unit patients in the United States during 1989-1999 Clin Infect Dis 2002 35 627 630 10.1086/342300 12173140

[B5] Blumberg HM Jarvis WR Soucie JM Edwards JE Patterson JE Pfaller MA Rangel-Frausto MS Rinaldi MG Saiman L Wiblin RT Wenzel RP National Epidemiology of Mycoses Survey (NEMIS) Study Group Risk factors for candidal bloodstream infections in surgical intensive care unit patients: the NEMIS prospective multicenter study. The National Epidemiology of Mycosis Survey Clin Infect Dis 2001 33 177 186 10.1086/321811 11418877

[B6] Leon C Ruiz-Santana S Saavedra P Almirante B Nolla-Salas J Alvarez-Lerma F Garnacho-Montero J Leon MA A bedside scoring system ("Candida score") for early antifungal treatment in nonneutropenic critically ill patients with Candida colonization Crit Care Med 2006 34 730 737 10.1097/01.CCM.0000202208.37364.7D 16505659

[B7] Leroy O Gangneux JP Montravers P Mira JP Gouin F Sollet JP Carlet J Reynes J Rosenheim M Regnier B Lortholary O AmarCand Study Group Epidemiology, management, and risk factors for death of invasive Candida infections in critical care: a multicenter, prospective, observational study in France (2005-2006) Crit Care Med 2009 37 1612 1618 10.1097/CCM.0b013e31819efac0 19325476

[B8] Pittet D Monod M Suter PM Frenk E Auckenthaler R Candida colonization and subsequent infections in critically ill surgical patients Ann Surg 1994 220 751 758 10.1097/00000658-199412000-00008 7986142 PMC1234477

[B9] Ostrosky-Zeichner L Sable C Sobel J Alexander BD Donowitz G Kan V Kauffman CA Kett D Larsen RA Morrison V Nucci M Pappas PG Bradley ME Major S Zimmer L Wallace D Dismukes WE Rex JH Multicenter retrospective development and validation of a clinical prediction rule for nosocomial invasive candidiasis in the intensive care setting Eur J Clin Microbiol Infect Dis 2007 26 271 276 10.1007/s10096-007-0270-z 17333081

[B10] Connor S Alexakis N Neal T Raraty M Ghaneh P Evans J Hughes M Rowlands P Garvey CJ Sutton R Neoptolemos JP Fungal infection but not type of bacterial infection is associated with a high mortality in primary and secondary infected pancreatic necrosis Dig Surg 2004 21 297 304 10.1159/000080884 15365228

[B11] Gotzinger P Wamser P Barlan M Sautner T Jakesz R Fugger R Candida infection of local necrosis in severe acute pancreatitis is associated with increased mortality Shock 2000 14 320 323 discussion 323-324 10.1097/00024382-200014030-00014 11028550

[B12] De Waele JJ Vogelaers D Blot S Colardyn F Fungal infections in patients with severe acute pancreatitis and the use of prophylactic therapy Clin Infect Dis 2003 37 208 213 10.1086/375603 12856213

[B13] Gloor B Muller CA Worni M Stahel PF Redaelli C Uhl W Buchler MW Pancreatic infection in severe pancreatitis: the role of fungus and multiresistant organisms Arch Surg 2001 136 592 596 10.1001/archsurg.136.5.592 11343553

[B14] King NK Siriwardana HP Wood B Siriwardena AK Trends in fungal colonization of pancreatic necrosis in patients undergoing necrosectomy for acute pancreatitis HPB (Oxford) 2005 7 120 123 10.1080/13651820510028837 18333174 PMC2023935

[B15] Vege SS Gardner TB Chari ST Baron TH Clain JE Pearson RK Petersen BT Farnell MB Sarr MG Outcomes of intra-abdominal fungal vs. bacterial infections in severe acute pancreatitis Am J Gastroenterol 2009 104 2065 2070 10.1038/ajg.2009.280 19491825

[B16] Eggimann P Francioli P Bille J Schneider R Wu MM Chapuis G Chiolero R Pannatier A Schilling J Geroulanos S Glauser MP Calandra T Fluconazole prophylaxis prevents intra-abdominal candidiasis in high-risk surgical patients Crit Care Med 1999 27 1066 1072 10.1097/00003246-199906000-00019 10397206

[B17] Faiz S Neale B Rios E Campos T Parsley E Patel B Ostrosky-Zeichner L Risk-based fluconazole prophylaxis of Candida bloodstream infection in a medical intensive care unit Eur J Clin Microbiol Infect Dis 2009 28 689 692 10.1007/s10096-008-0666-4 19011913

[B18] Garbino J Lew DP Romand JA Hugonnet S Auckenthaler R Pittet D Prevention of severe Candida infections in nonneutropenic, high-risk, critically ill patients: a randomized, double-blind, placebo-controlled trial in patients treated by selective digestive decontamination Intensive Care Med 2002 28 1708 1717 10.1007/s00134-002-1540-y 12447512

[B19] Pelz RK Hendrix CW Swoboda SM Diener-West M Merz WG Hammond J Lipsett PA Double-blind placebo-controlled trial of fluconazole to prevent candidal infections in critically ill surgical patients Ann Surg 2001 233 542 548 10.1097/00000658-200104000-00010 11303137 PMC1421284

[B20] Mean M Marchetti O Calandra T Bench-to-bedside review: Candida infections in the intensive care unit Crit Care 2008 12 204 10.1186/cc6212 18279532 PMC2374590

[B21] Nathens AB Curtis JR Beale RJ Cook DJ Moreno RP Romand JA Skerrett SJ Stapleton RD Ware LB Waldmann CS Management of the critically ill patient with severe acute pancreatitis Crit Care Med 2004 32 2524 2536 10.1097/01.CCM.0000148222.09869.92 15599161

[B22] Scoring systems for ICU and surgical patients: APACHE II http://www.sfar.org/score2/apache22.html

[B23] Levy MM Fink MP Marshall JC Abraham E Angus D Cook D Cohen J Opal SM Vincent JL Ramsay G 2001 SCCM/ESICM/ACCP/ATS/SIS International Sepsis Definitions Conference Crit Care Med 2003 31 1250 1256 10.1097/01.CCM.0000050454.01978.3B 12682500

[B24] DeLong ER DeLong DM Clarke-Pearson DL Comparing the areas under two or more correlated receiver operating characteristic curves: a nonparametric approach Biometrics 1988 44 837 845 10.2307/2531595 3203132

[B25] Grewe M Tsiotos GG Luque de-Leon E Sarr MG Fungal infection in acute necrotizing pancreatitis J Am Coll Surg 1999 188 408 414 10.1016/S1072-7515(98)00334-2 10195725

[B26] Hoerauf A Hammer S Muller-Myhsok B Rupprecht H Intra-abdominal Candida infection during acute necrotizing pancreatitis has a high prevalence and is associated with increased mortality Crit Care Med 1998 26 2010 2015 10.1097/00003246-199812000-00031 9875913

[B27] Isenmann R Schwarz M Rau B Trautmann M Schober W Beger HG Characteristics of infection with Candida species in patients with necrotizing pancreatitis World J Surg 2002 26 372 376 10.1007/s00268-001-0146-9 11865377

[B28] Kochhar R Ahammed SK Chakrabarti A Ray P Sinha SK Dutta U Wig JD Singh K Prevalence and outcome of fungal infection in patients with severe acute pancreatitis J Gastroenterol Hepatol 2009 24 743 747 10.1111/j.1440-1746.2008.05712.x 19220667

[B29] Kumar A Roberts D Wood KE Light B Parrillo JE Sharma S Suppes R Feinstein D Zanotti S Taiberg L Gurka D Kumar A Cheang M Duration of hypotension before initiation of effective antimicrobial therapy is the critical determinant of survival in human septic shock Crit Care Med 2006 34 1589 1596 10.1097/01.CCM.0000217961.75225.E9 16625125

[B30] Playford EG Lipman J Kabir M McBryde ES Nimmo GR Lau A Sorrell TC Assessment of clinical risk predictive rules for invasive candidiasis in a prospective multicentre cohort of ICU patients Intensive Care Med 2009 35 2141 2145 10.1007/s00134-009-1619-9 19756510

[B31] Chakrabarti A Rao P Tarai B Shivaprakash MR Wig J Candida in acute pancreatitis Surg Today 2007 37 207 211 10.1007/s00595-006-3371-x 17342358

[B32] Gudlaugsson O Gillespie S Lee K Vande Berg J Hu J Messer S Herwaldt L Pfaller M Diekema D Attributable mortality of nosocomial candidemia, revisited Clin Infect Dis 2003 37 1172 1177 10.1086/378745 14557960

[B33] Marchetti O Bille J Fluckiger U Eggimann P Ruef C Garbino J Calandra T Glauser MP Täuber MG Pittet D Fungal Infection Network of Switzerland Epidemiology of candidemia in Swiss tertiary care hospitals: secular trends, 1991-2000 Clin Infect Dis 2004 38 311 320 10.1086/380637 14727199

[B34] León C Ruiz-Santana S Saavedra P Galván B Blanco A Castro C Balasini C Utande-Vázquez A González de Molina FJ Blasco-Navalproto MA López MJ Charles PE Martín E Hernández-Viera MA Cava Study Group Usefulness of the "Candida score" for discriminating between Candida colonization and invasive candidiasis in non-neutropenic critically ill patients: a prospective multicenter study Crit Care Med 2009 37 1624 1633 10.1097/CCM.0b013e31819daa14 19325481

[B35] Sobel JD Candida infections in the intensive care unit Crit Care Clin 1988 4 325 344 3048590

[B36] Ridley S Cardiac scoring systems--what is their value? Anaesthesia 2003 58 985 991 10.1046/j.1365-2044.2003.03342.x 12969039

[B37] Hermsen ED Zapapas MK Maiefski M Rupp ME Freifeld AG Kalil AC Validation and comparison of clinical prediction rules for invasive candidiasis in intensive care unit patients: a matched case-control study Crit Care 2011 15 R198 10.1186/cc10366 21846332 PMC3387640

